# Are Obese Patients with Autism Spectrum Disorder More Likely to Be Selenium Deficient? Research Findings on Pre- and Post-Pubertal Children

**DOI:** 10.3390/nu12113581

**Published:** 2020-11-22

**Authors:** Anna Błażewicz, Iwona Szymańska, Wojciech Dolliver, Piotr Suchocki, Jadwiga Turło, Agata Makarewicz, Katarzyna Skórzyńska-Dziduszko

**Affiliations:** 1 Department of Analytical Chemistry, Medical University of Lublin, 20-093 Lublin, Poland; iwonka.szyms@gmail.com; 2 The Division of Pulmonary and Critical Care Medicine, Brigham and Women’s Hospital, Boston, MA 02115, USA; wdolliver@bwh.harvard.edu; 3 Department of Bioanalysis and Drugs Analysis, Medical University of Warsaw, 02-793 Warsaw, Poland; piotr.suchocki@wum.edu.pl; 4 Department of Drug Technology and Pharmaceutical Biotechnology, Medical University of Warsaw, 02-793 Warsaw, Poland; jadwiga.turlo@wum.edu.pl; 5 Department of Psychiatry, Psychotherapy and Early Intervention, Medical University of Lublin, 20-439 Lublin, Poland; chemistry_coordinator@umlub.pl; 6 Department of Human Physiology, Medical University of Lublin, 20-080 Lublin, Poland; katarzyna.skorzynska-dziduszko@umlub.pl

**Keywords:** obesity, autism, selenium, thyroid hormones

## Abstract

Selenium is involved in many metabolic pathways that are critical for life. Information concerning the metabolic effects of selenium in autism spectrum disorder (ASD) and obesity is still conflicting and incomplete. The pre- and post-pubertal selenium profiles of patients with ASD and obesity have not yet been investigated. The goal of the study was to examine selenium content before and after puberty in euthyroid children diagnosed with ASD, compared to age-matched neurotypical controls, with respect to overweight or obesity as a co-existing pathology. Serum, toenail, and 24h urine selenium levels were determined by inductively coupled plasma mass spectrometry in 287 prepubertal children (mean age 8.09 years), divided into groups: ASD with overweight/obesity (ASD+/Ob+); ASD without overweight/obesity (ASD+/Ob−); non-ASD with overweight/obesity (ASD−/Ob+); and non-ASD without overweight/obesity (ASD−/Ob−). The assessment was repeated in 258 of the children after puberty (mean age 14.26 years).The lowest serum (*p* < 0.001), urine (*p* < 0.001) and toenail (*p* < 0.001) selenium levels before and after puberty were observed in ASD+/Ob+ patients, and the highest in ASD−/Ob−. There were no differences in serum/toenail selenium levels between ASD+/Ob− and ASD−/Ob+ groups. The presence of ASD was associatedwith lower serum (*p* < 0.001) and toenail (*p* < 0.001) selenium in BMI-matched groups. In neurotypical patients, post-pubertal serum selenium levels were lower (*p* < 0.001) than pre-pubertal levels. In the multiple linear regression analyses, selenium levels showed inverse relationships with BMI (*p* < 0.001) and male gender (*p* < 0.001), irrespective of the sample type. The serum (*p* = 0.002) and toenail (*p* < 0.001) selenium levels were inversely associated with the presence of ASD. ASD, obesity/overweight, and male gender have independent impacts on selenium levels in children. Puberty may affect selenium content in neurotypical children of both genders, but not in ASD patients.

## 1. Introduction

Selenium (Se) is an essential nutrient and a component of many seleno-proteins having critical roles in thyroid function, fetal development, hormone metabolism, and oxidative stress detoxification, particularly in endocrine and brain tissues. Many redox regulatory mechanisms that are required for healthy cell growth and function may be disrupted by Se-dependent metabolic processes. Although the role of selenium in health and disease has been debated for many years, there is no information on possible changes in the selenium profile of patients with ASD and obesity during puberty. ASD includes several disorders (autistic disorder, pervasive developmental disorder not otherwise specified (PDD-NOS), and Asperger syndrome (AS)), with a wide range of severity of symptoms and behaviors and considerable individual variation [1,2]. Research on the etiology of autism faces a major challenge due to the complex interplay between environmental, genetic, and social factors [3]. Results vary significantly from study to study, and unfortunately there have been no repeated studies on the same population carried out under identical conditions [4,5,6].

Existing evidence indicates that patients with ASD have lower selenium status, greateroxidative stress manifesting as increased peroxidation of lipid biomolecules, and decreased methylation capacity [7,8]. Several studies have reported that some individuals with ASD express polymorphisms in genes involved in the detoxification of environmental pollutants, such as endocrine disrupting chemicals (EDCs) including heavy metals [9]. Additionally, disturbances in detoxification have often been highlighted in autism [10,11].

Both autism and obesity affect a growing population around the world, and there is no indication that this unfavorable global trend will change in the near future [12,13].

Studies have found that children and adolescents with neurodevelopmental problems are at higher risk of obesity [14,15,16].The problem of obesity in autism should not be marginalized, and further research is needed to study factors associated with obesity in the population with autism spectrum disorders (ASD) [17]. Furthermore, information concerning the metabolic effects of selenium in obesity is still conflicting and incomplete, and therefore studies on the health effects of selenium in obesity are of considerable interest.

As our previous research [18] showed that obesity significantly influences the level of Se, and given the important role of Se in the endocrine system and increasing evidence of the significant role of Se in autism, we decided to investigate possible changes in Se content in euthyroid individuals diagnosed with ASD compared to neurotypical people. The participants were tested during two developmental stages, i.e., childhood and early adulthood (before and after adolescence). An additional goal was to check whether and to what extent obesity and autism, as complex, multifactorial pathologies affecting the whole body and influencing numerous aspects of human function, affect the selenium level in the body.

We undertook a long-term (before and after puberty) research project, which involved measurement of selenium levels in various tissues, i.e., serum (reflecting recent daily intake), and toenails (as a long-term indicator of Se status and reflecting differences in Se intake over a longer period), as well as in urine (reflecting recent exposure).

## 2. Materials and Methods

### 2.1. Study and Control Populations

The study was carried out in accordance with the Code of Ethics of the World Medical Association (Declaration of Helsinki, as revised in 2013) for experiments involving humans. The study protocol was reviewed and approved by the Ethics Committee of the Medical University of Lublin (KE-0254/12/2014).

The study was conducted on a carefully chosen population diagnosed with ASD. We considered only the diagnosis, and not the severity of the disorder. The diagnosis was based on the criteria for autistic disorder as defined in the Diagnostic and Statistical Manual of Mental Disorders, Fifth Edition (DSMV), Autism Diagnostic Interview Revised (ADI-R) and Autism Rating Scale (CARS) [2]. Inclusion criteria included prepubertal age and ASD diagnosis. Patients were recruited through local support groups (mainly parents and caregivers) or were referred by specialist clinicians and therapists. The informed consent of the children’s parents or legal guardians was obtained.

The control group (neurotypical children and adolescents) was selected from among children visiting the medical center for regularly scheduled check-ups. They were not receiving any medical treatment and were not admitted to the hospital. All members of the control and study groups were from the same geographic area (central and south-eastern Poland).

Neither the control nor the ASD group presented pathologies of the thyroid gland, and they had taken no mineral or vitamin supplements for at least three months before the samples for analysis were collected. They were not on any special diets during the tests. Other exclusion criteria included the presence of a chronic condition (particularly affecting weight or limiting the patient’s ability to participate in the study), drug treatment for any acute or chronic condition, and refusal to give informed consent.

An initial personal interview was conducted by trained personnel to establish household characteristics, dietary behavior, health status, and socioeconomic characteristics. A child’s weight status was determined using an age- and sex-specific percentile for body mass index (BMI, in kg/m^2^). Current and representative BMI percentile charts for the Polish population of children and adolescents (3–18 year of age) were used [19]. BMI was divided into two categories representing normal weight or elevated weight (both overweight and obesity). According to the Polish percentile charts, a BMI above the 85thpercentile (+1 SD) for age and gender was considered overweight [19].

**Figure 1:**
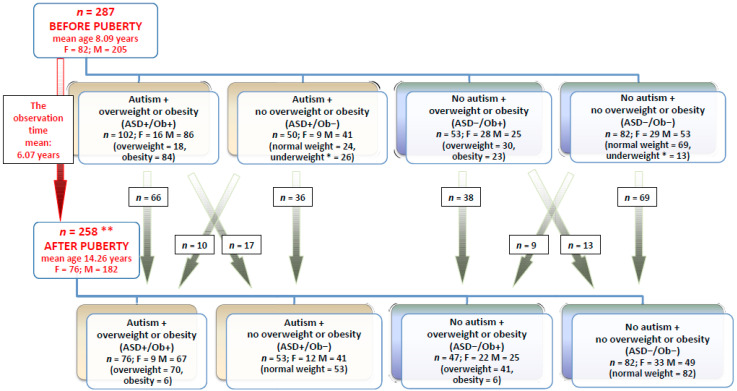
Allocation of pre- and post-pubertal children to groups. F—female, M—male. * Children with mild underweight (near-normal weight) who presented normal physical development and normalized weight after puberty (see below). ** A total of 29 children did not participate in the study after puberty.

A total of 287 prepubertal children (mean age 8.09 years, SD 1.36) were recruited into the study and divided into four groups: autistic patients with overweight or obesity (ASD+/Ob+); autistic patients without overweight or obesity (ASD+/Ob−); non-autistic patients with overweight or obesity (ASD−/Ob+); and non-autistic patients without overweight or obesity (ASD−/Ob−). The clinical assessment was repeated after normal puberty in a total of 258 children (mean age 14.26 years, SD 1.37) in the four groups (Figure 1). The study combined data from the 2014/2015 and 2019/2020 cycles of measurements to provide more statistically reliable estimates.

Children were diagnosed with underweight (*n* = 39) according to the above mentioned BMI percentile charts (for age and gender) for the Polish population of children and adolescents [19]. BMI values below −1 SD for female as well as BMI values below a half, between −1 SD and −2 SD, for male were considered underweight.

The prepubertally underweight male group (*n* = 34; 24 boys from the ASD+/Ob−group; 10 boys from the ASD−/Ob−group) was characterized as follows:

1/ the mean BMI 13.42 kg/m^2^; standard deviation 0.69; median 13.53 kg/m^2^; interquartile range 0.9; minimal value 11.9 kg/m^2^; maximal value 14.69 kg/m^2^;

2/ the mean age 7.39 years; standard deviation 1.14; median 7.92 kg/m^2^; interquartile range 2.0; minimal value 6 years; maximal value 10 years.

According to the Polish BMI percentile chart underweight in a male is considered below 13.8 kg/m^2^ for age 7.39 years.

The prepubertally underweight female group (*n* = 5; 1 girl from the ASD+/Ob− group; 4 girls from the ASD−/Ob− group) was characterized as follows:

1/ the mean BMI 13.4 kg/m^2^; standard deviation 0.74; median 13.23 kg/m^2^; interquartile range 1.11; minimal value 12.6 kg/m^2^; maximal value 14.35 kg/m^2^;

2/ the mean age 7.15 years; standard deviation 1.01; median 7.83 kg/m^2^; interquartile range 1.75; minimal value 6 years; maximal value 8 years.

According to the Polish BMI percentile charts underweight in a female is considered below 14 kg/m^2^ for age 7.15 years.

All pre-pubertally underweight children presented normal physical development pre- and post-pubertally, as well as they spontaneously normalized weight after puberty. The mean difference between the normal BMI cut-offs (stratified for age and gender) and BMI calculated in the underweight group was 0.43 kg/m^2^. Therefore, we decided to include this group in the study. The single outliers in the data were as follows: BMI 11.96 kg/m^2^ for the 6-years old boy (the ASD+/Ob−group) and BMI 11.9 kg/m^2^ for the 6-years old boy (the ASD−/Ob−group).

### 2.2. Samples and Their Analyses

Measurements of serum-free triiodothyronine (fT3), free thyroxine (fT4) and thyroid stimulating hormone (TSH) were performed by accredited diagnostic labs. Blood samples from the cubital vein were collected in the morning after overnight fasting. Twenty-four-hour urine samples were collected for three consecutive days. Toenail samples were collected on the same day as the body fluid tests. The detailed procedures of serum, urine and nail sample collection, storage, preparation, and inductively coupled plasma mass spectrometry (ICP-MS) analysis were described in our previous papers [20,21,22].

The study material collected during the two assessment periods consisted of 555 samples of serum, 555 samples of urine, and 555 samples of toenails. Samples taken from all the groups (Figure 1) were pre-treated and analyzed in the same way.

### 2.3. Statistical Analyses

The required sample sizes were calculated using G*Power v3.1.7 (Heinrich Heine Universität Düsseldorf, Düsseldorf, Germany) with 80% power (alpha = 0.05, two-tailed) as a part of the research planning process.

Descriptive statistics were produced for the overall sample and also stratified by both autism and BMI status. As the Kolmogorov-Smirnov and Lilliefors tests indicated that the variables were not normally distributed, the nonparametric Kruskal-Wallis ANOVA test was used for continuous variables. The Wilcoxon signed-rank test was used to compare related samples before and after puberty. The Spearman’s correlation coefficient test was used to measure relationships between the variables. A multiple linear regression analysis was used to evaluate the association between the serum selenium, urine selenium, and nail selenium levels and ASD status, BMI, and gender. Logistic regression analysis was performed to estimate the odds ratios and 95% confidence intervals of ASD status by BMI as well as the serum selenium, urine selenium, and nail selenium levels, adjusted for potential confounders. All analyses were two-tailed with a significance level of 0.05. Statistical analyses were performed using TIBCO Software Inc. (2017) Statistica, v 13.0.0.0 (TIBCO, Tulsa, USA). The program licensed to the Medical University of Lublin (used by Katarzyna Skórzyńska-Dziduszko).

## 3. Results

A detailed characterization of children divided into four groups is presented in Table 1. As the variables were not normally distributed, and the nonparametric median tests were used for the comparison of samples, the mean and standard deviation values are presented in Table 1 only for the full characterization of study groups.

All the groups were age-matched before and after puberty. There were no significant differences in TSH or free thyroid hormone (fT4 and fT3) levels between the groups stratified by ASD and BMI. Furthermore, there were no significant differences in BMI between the ASD+/Ob+ and ASD−/Ob+ groups or between the ASD+/Ob− and ASD−/Ob−groups.

In the extended analysis of the subgroups stratified for BMI (obesity versus overweight or normal weight versus borderline underweight), there were no significant differences in age, TSH, fT4, fT3, or selenium levels in the ASD and non-ASD subgroups.

### 3.1. Selenium in Serum

**Figure 2:**
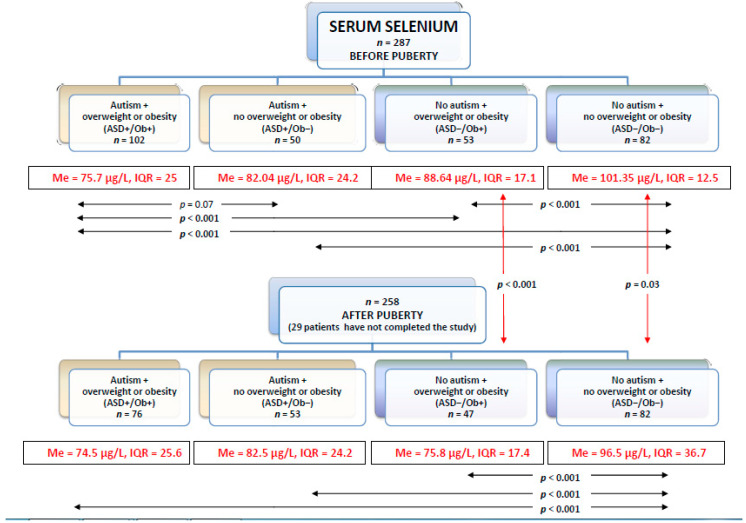
The serum selenium levels in each group before and after puberty (Me—median, IQR—interquartile range).

The serum selenium levels in the groups before and after puberty are presented in Figure 2.

The lowest serum selenium levels before and after puberty were observed in the ASD+/Ob+ patients, while the highest were noted in the healthy controls with normal body weight(ASD−/Ob−). Interestingly, there were no significant differences in the serum selenium levels between the ASD+/Ob− and ASD−/Ob+ patients.

Before puberty, serum selenium levels were lower in the ASD+/Ob+ patients than in the ASD−/Ob+ group, but no significant differences were observed after puberty. Among patients without overweight or obesity, the ASD+/Ob− group presented lower serum selenium levels than the ASD−/Ob− group, and the difference was significant before and after puberty. The presence of ASD was associated with lower serum selenium levels in the BMI-matched groups of patients without overweight or obesity.

Puberty had no impact on serum selenium levels in the ASD patients, whereas in the neurotypical patients post-pubertal serum Se levels were significantly lower than pre-pubertal levels. However, in the extended analysis of the subgroups stratified for BMI (with respect to the direction of BMI changes presented by children after puberty in comparison to their pre-pubertal BMI), one group of patients (ASD children with pre-pubertal overweight or obesity but normalized BMI after puberty, *n* = 17) presented significantly lower (*p* = 0.003) pre-pubertal serum selenium levels (median 76.9 μg/L, interquartile range 29) than the post-pubertal children (median 98 μg/L, interquartile range 10.5).

Before and after puberty, lower serum selenium levels were observed in the ASD+/Ob+ patients than in the ASD−/Ob+ patients. The same applied to patients without overweight or obesity—the ASD group with normal BMI presented lower serum selenium levels than the non-ASD group. The presence of ASD was associated with lower serum selenium levels in the BMI-matched groups. Furthermore, the presence of overweight or obesity was associated with lower serum selenium levels in the ASD-matched groups of patients.

### 3.2. Selenium in Urine

**Figure 3:**
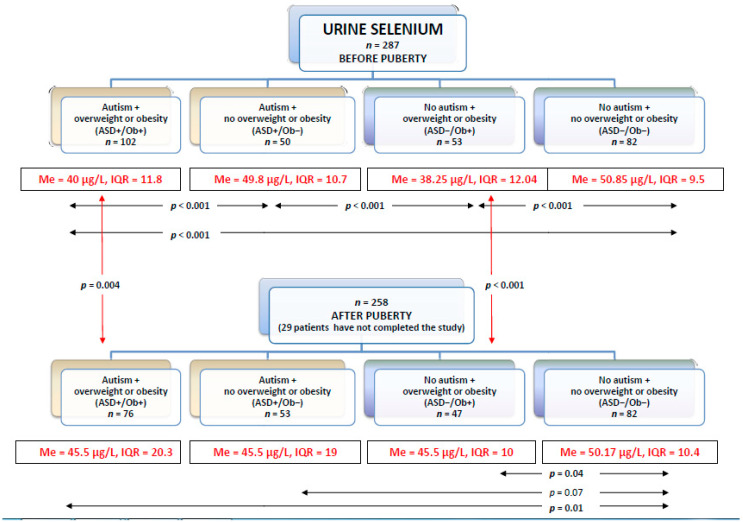
24 h urinesample selenium levels in each group before and after puberty (Me—median, IQR—interquartile range).

The urine selenium levels in the groups before and after puberty are presented in Figure 3.

Both before and after puberty the highest urine selenium levels were observed in the ASD−/Ob− group. The finding was concordant with the highest serum selenium levels observed in this group.

In patients with overweight or obesity, there were no differences between patients with or without an ASD diagnosis in either pre-pubertal or post-pubertal urine selenium levels. Interestingly, urine selenium levels in these groups were significantly higher after puberty than before its onset. This difference was not seen in either group of patients without overweight/obesity (with ASD or neurotypical).

### 3.3. Selenium in Toenails

**Figure 4:**
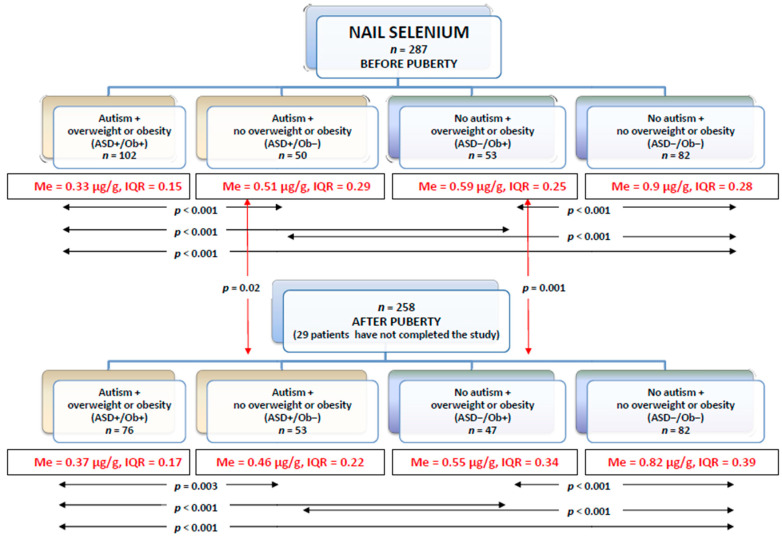
Toenail selenium levels in each group before and after puberty (Me—median, IQR—interquartile range).

The toenail selenium levels in each group before and after puberty are presented in Figure 4.

Before and after puberty, the lowest toenail selenium levels were observed in the ASD+/Ob+ patients, while the highest levels were detected in the ASD−/Ob− group. As in the case of the serum selenium levels, there were no significant differences in toenail selenium levels between the ASD+/Ob−and ASD−/Ob+ patients.

### 3.4. The Spearman’s Correlations between Serum, Urine and Toenail Selenium Concentrations

Table 2 and Table 3 present the results of the Spearman’s rank-order correlation tests. The serum, urine and toenail selenium levels showed significant positive inter-correlations before and after puberty.

### 3.5. Association between Serum, Urine, and Toenail Se Levels and ASD Status, BMI, and Gender

In the multiple linear regression analyses (Table 4), the serum, urine and toenail selenium levels showed significant inverse relationships with BMI and male gender. The serum and toenail selenium levels (but not the selenium urine levels) were significantly inversely associated with the presence of ASD. Logistic regression models showed that the presence of ASD was associated with decreased odds of both serum and toenail concentrations of selenium in the model adjusted for age, gender and BMI. Obesity/overweight was associated with decreased odds of both serum and toenail selenium concentrations in the model adjusted for age, gender and ASD status. Male gender was associated with decreased odds of selenium serum, urine and toenail concentrations in the model adjusted for age, as well as ASD and obesity/overweight statuses.

In conclusion, ASD, obesity/overweight, and male gender have independent and significant impacts on selenium levels in children.

## 4. Discussion

It is well established that adequate levels of selenium are important for the functioning of the central nervous system, endocrine system, reproductive system, and cardiovascular system, as well as for regulating immune processes [23]. A very recent review of the literature on Se metabolism in adipocyte physiology and obesity pathogenesis concluded that Se appears to play an essential role in adipose tissue physiology and pathophysiology, but strongly emphasized the huge inconsistency in the findings of studies on the human population [23].

It has already been documented that increased BMI can be considered a significant predictor of blood selenium deficiency and low selenium intake [24]. Our findings on the reduced serum and toenail selenium levels in obese/overweight children generally corroborate these data.

Studies have found that the prevalence of excessive weight is significantly greater among children with ASD compared with the general population [15], and that children and adolescents with neurodevelopmental problems are at higher risk of obesity [16]. Moreover, the results have shown significantly raised prevalence rates of ASD in populations with eating disorders compared with healthy control participants [25]. Children with autism often present higher food selectivity, which may result in a variety of nutritional deficiencies, including selenium deficiency. A recent meta-analysis reported that the dietary pattern in children with ASD is characterized by lower intake of Se in comparison to controls [26].

The mechanisms that link obesity, ASD and selenium deficiency remain unclear. An excess of adipose tissue, which results from chronic over-nutrition, leads to immune and inflammatory responses of white adipose tissue, contributing to systemic chronic low grade inflammation, frequently referred to as a metabolic inflammation [27].

In recent years huge progress has been made in identifying the mechanisms by which selenium modifies the inflammatory response in murine cell lines as well as in animal models of knockout mice [28,29,30]. The available data strongly suggest that selenium mitigates the inflammatory response, but the conclusions do not directly explain whether reduced selenium body stores, as observed in our study in obese children, may enhance obesity-associated inflammation, which in turn could promote the development and progression of obesity. There is a great deal of scientific evidence that the interplay of the gut microbiota with the brain affects autism through the neuro–endocrine–immune network [31,32], and some animal studies suggest that gut bacteria may compete with the host for selenium when its availability in the organism becomes limited [33]. It is postulated that individuals with ASD show intestinal dysbiosis characterized by the imbalance between beneficial microbes and gut pathogenic microbes resulting in an increased presence of toxic molecules affecting the neurotransmitter function in the brain, which may lead to abnormalities in behavioral patterns [34]. However, to date there is no strong confirmation of the role of Se in modulating the microbiota during inflammation in humans. A review of the research on the interactions between Se metabolism and ASD and on the role of Se in inhibiting oxidative stress, neuroinflammation, microglia activation, excitotoxicity, synapse dysfunction, and gut–brain axis disturbance does not allow for consistent conclusions confirming the neuroprotective effects of Se in all individuals with ASD [35]. Many studies highlight increased levels of heavy metals, disturbances in detoxification, and impaired methylation and redox homeostasis with increased vulnerability to oxidative stress in ASD [7,8,9,10,11], thus it cannot be excluded that at least a partial decrease of Se content in ASD may be due to its ability to form compounds with metals toxic to humans. Research has showed that, e.g., mercury has high affinity to Se and it was found that a mercury-induced selenium deficiency state inhibits regeneration of the seleno-proteins. Moreover, it was suggested that the effect of mercury is to produce a selenium deficiency state and a direct inhibition of selenium’s role in controlling the intracellular redox environment in organisms [36].

According to our study, males present lower selenium concentrations than females. It has recently been observed that inadequate consumption of selenium by Mexican children may be associated with later pubertal development in boys, but not in girls, suggesting a sex-specific pattern [37]. However, the results of this research cannot be applied to our study, since all children presented normal pubertal development, and the selenium serum/urine/toenail levels did not differ before and after puberty in ASD patients of either gender (data not shown). Preliminary data from a mouse model suggest that the brain and testes may compete for selenium utilization, with concomitant effects on neurodevelopment and neurodegeneration [38]. In our study, puberty seemed to affect the selenium balance only in neurotypical children of both genders. Since there are no available data that could help to explain these discrepancies, future work with humans and measures of selenium biomarkers is needed for a better understanding of the role of selenium in children’s sexual development.

The reference values of serum Se established for healthy individuals above the age of one year amount to 70–150 ng/mL [39], and our control group (ASD−/Ob−) fell within this range in both periods of testing. More or less decreased levels of Se are reported in tissues of ASD individuals, whether the studies analyzed serum, red blood cells, or nails [5,40,41]. These findings generally corroborate our results, which demonstrate that the serum and toenail selenium levels are significantly inversely associated with the presence of ASD.

Interestingly, in our study euthyroid TSH/fT4/fT3 serum concentrations were not associated with serum/urine/toenail selenium levels, nor were there any differences in the levels of these hormones between the groups of patients stratified by ASD. None of the patients presented thyroid gland pathology, which may explain the lack of associations between the normal thyro-metabolic state and selenium levels, altered by obesity or ASD co-incidence.

The liver regulates whole-body Se distribution by sorting metabolically available Se between the two pathways of Se-protein synthesis and excretory metabolite synthesis [42]. The fraction of Se that cannot be utilized for Se-protein synthesis enters the excretory pathway. Urine selenium levels reflect excretory pathway involvement, which is dynamically altered to maintain selenium homeostasis. This dynamic alteration may explain the weak correlations between urine selenium levels and toenail selenium concentrations.

### Advantages and Limitations of the Study

A limited number of studies demonstrate Se problems in obesity and autism, whether the two disorders are considered together or separately. Moreover, there is a lack of research measuring selenium in the population with both obesity and ASD during more than one period of time. In our research, conducted during two different stages of biological development, we not only observed differences between and within groups, but also identified important changes in the Se level over time. We measured the same variables for the same group of people according to identical criteria. The criteria for inclusion in the study were kept constant, and the study groups were matched for age and gender to the control group. All study participants were from the same geographic area (human selenium levels show marked geographic variation, depending on dietary intake) [43].

Measurement of selenium content in three significantly different biological matrices (serum, urine, and toenails) proved to significantly influence the results. Although serum concentrations of Se provide useful information in the clinical categorization of deficiency and toxicity states [44], this type of sample has several disadvantages—associated with collection, transport, and storage—over toenail material, which is an easily accessible and long-term stable source of Se and an indicator of environmental exposure [45].

The biological effects, toxicity and bioavailability of Se may strongly depend on its chemical form. We were not able to study these forms due to sample pre-treatment requirements, and therefore our determinations refer to the total Se content in a given sample.

We have not yet described the intensity of the ASD symptoms presented by the patients and their association with selenium levels and obesity. An additional complement to future studies may examine the level of sex hormones in the obese and ASD populations since elevated levels of prenatal androgens such as fetal testosterone during a critical period are hypothesized to contribute to the etiology of autism spectrum [46]. Hormonal abnormalities and an increased rate of higher testosterone-related medical conditions have been reported in women with autism spectrum [47,48].

Obesity or overweight is typically the first stimulus prompting dietary intervention, and many individuals with autism are on various specific diets (e.g., a gluten-free, casein-free or low-fat diet) [49], and thus a thorough analysis of selenium status in relation to nutrition and diets could complement our future research. Lifestyle factors affecting choice of diet and physical activity, as well as the type of pharmacological treatment, are issues for further study that may help to determine whether Se imbalance is mainly due to altered metabolic processes, insufficient dietary intake, or both.

## 5. Conclusions

Our study confirms a complex interplay between selenium imbalance, obesity and ASD. The findings suggest that puberty affects selenium content in neurotypical children of both genders. Serum/urine/toenail Se levels did not differ before and after puberty in ASD patients of either gender.

The mechanisms that link selenium imbalance to adipose tissue pathophysiology or the presence of ASD have yet to be fully elucidated. Recent data from both human and laboratory studies on the role of Se in obesity and adipose tissue function are highly heterogeneous [23]. Further human and laboratory studies are required to highlight the association between Se intake and obesity. An interesting question arises as to whether reduced selenium plays a causative role in the development of ASD and obesity or is only one of the biomarkers of the wide variety of nutritional imbalances that develop in the course of these pathologies. Therefore, the biological role of selenium in the development of ASD should be a subject of future investigations, which may at some point produce results applicable to strategies for the management of the disorder.

## Figures and Tables

**Table 1:** Characteristics of pre- and post-pubertal children.

Variables	Mean	Median	Min.	Max.	IQR	SD	Mean	Median	Min.	Max.	IQR	SD
	Before Puberty						After Puberty					
**ASD Patients with Overweight or Obesity (ASD+/Ob+)**												
Age (years)	7.98	8.17	6.0	10.58	2.79	1.46	14.07	14.25	12.08	16.67	2.71	1.46
BMI (kg/m^2^)	24.8	25.51	13.21	35.16	4.96	4.97	25.96	25.7	22.66	31.14	2.32	1.83
TSH (µIU/mL)	2.67	2.94	0.63	4.92	1.16	0.99	3.14	3.15	0.66	5.21	2.18	1.3
fT4 (pmol/L)	16.18	15.52	10.07	29.9	6.35	4.31	15.54	15.46	9.85	25.68	4.59	3.72
fT3 (pmol/L)	4.83	4.55	3.55	6.95	1.03	0.84	5.03	4.99	3.78	8.12	1.26	0.89
Se serum (µg/L)	72.99	75.7	44.8	99.1	25.0	14.04	74.89	74.5	52.5	115.6	25.59	15.32
Seurine (µg/L)	41.16	40.0	25.3	69.7	11.8	9.5	45.38	45.5	22.15	70.2	20.3	12.42
Se toenails (µg/g)	0.35	0.33	0,12	0.74	0.15	0.01	0.38	0.37	0.09	0.82	0.13	0.13
**ASD Patients Without Overweight or Obesity (ASD+/Ob−)**												
Age (years)	7.96	8.17	6.0	10.33	2.33	1.39	14.09	14.25	12.08	17.0	2.42	1.42
BMI (kg/m^2^)	18.31	16.15	11.96	31.99	9.18	6.01	20.47	20.66	16.76	23.11	2.15	1.6
TSH (µIU/mL)	2.17	2.1	0.54	4.74	2.0	1.27	1.27	0.99	0.45	3.2	0.68	0.69
fT4 (pmol/L)	17.2	17.44	11.2	25.5	6.9	4.02	19.43	19.8	10.22	26.5	5.06	3.91
fT3 (pmol/L)	5.32	4.96	4.15	7.11	1.71	0.97	5.78	5.6	3.99	8.11	1.72	1.04
Se serum (µg/L)	80.86	82.04	54.1	105	24.16	14.36	80.98	82.5	52.56	112.55	24.63	15.68
Se urine (µg/L)	51.68	49.8	31.5	80.55	10.75	11.68	46.53	45.5	21.56	79.5	19.0	13.79
Se toenails (µg/g)	0.52	0.51	0.13	0.94	0.29	0.19	0.51	0.46	0.25	0.94	0.22	0.17
**Non-ASD Patients with Overweight or Obesity (ASD−/Ob+)**												
Age (years)	8.06	7.5	6.08	10.5	2.0	1.31	14.08	13.5	12.17	16.5	2.17	1.26
BMI (kg/m^2^)	21.62	22.7	13.77	27.77	2.4	3.79	25.89	25.46	22.19	30.67	2.18	2.07
TSH (µIU/mL)	2.65	2.5	0.98	4.62	1.33	0.98	2.98	2.89	1.07	4.99	1.16	0.85
fT4 (pmol/L)	14.48	14.66	9.32	18.66	2.92	2.06	15.68	15.5	11.15	24.55	2.08	2.57
fT3 (pmol/L)	5.07	4.99	3.95	6.78	1.01	0.75	5.17	5.12	4.26	6.71	1.04	0.68
Se serum (µg/L)	86.14	88.64	57.72	119.3	17.14	14.37	78.17	75.85	54.8	126.6	17.45	13.44
Se urine (µg/L)	40.01	38.25	23.97	59.5	12.04	8.87	45.93	45.5	32.5	65.8	10.0	6.97
Se toenails (µg/g)	0.66	0.6	0.32	1.45	0.25	0.21	0.57	0.55	0.17	0.98	0.34	0.23
**Non-ASD Patients Without Overweight or Obesity (ASD−/Ob−)**												
Age (years)	8.6	8.54	6.0	10.83	1.5	1.24	14.6	14.67	12.08	16.83	1.5	1.24
BMI (kg/m^2^)	17.05	16.52	11.9	23.99	3.12	2.54	20.73	20.66	18.7	23.46	1.83	1.22
TSH (µIU/mL)	1.39	1.1	0.5	3.98	1.25	0.71	1.61	1.47	0.17	3.87	1.41	0.89
fT4 (pmol/L)	16.6	16.47	11.6	24.45	4.63	2.92	17.1	16.54	11.25	24.25	5.1	3.11
fT3 (pmol/L)	5.43	5.28	4.01	6.74	1.19	0.73	5.35	5.23	4.2	6.7	1.0	0.66
Se serum (µg/L)	104.93	101.35	66.3	149.5	12.5	15.27	98.8	96.52	62.48	145.2	36.68	20.66
Se urine (µg/L)	52.36	50.85	38.1	69.5	9.5	7.2	50.54	50.17	32.5	89.6	10.4	8.67
Se toenails (µg/g)	0.97	0.9	0.24	1.96	0.28	0.34	0.86	0.82	0.4	1.92	0.39	0.33

BMI—body mass index; min—minimal value; max—maximal value; IQR—interquartile range; SD—standard deviation.

**Table 2:** Inter-correlations between age, BMI, thyroid stimulating hormone (TSH), fT3, fT4 and serum, urine and toenail selenium concentrations. Statistically significant correlations are highlighted. Spearman’s rank-order correlation test results.

Variablesi	Before Puberty					After Puberty				
	Total	ASD+/Ob+	ASD+/Ob−	ASD−/Ob+	ASD−/Ob−	Total	ASD+/Ob+	ASD+/Ob−	ASD−/Ob+	ASD−/Ob−
	*n* = 287	*n* = 102	*n* = 50	*n* = 53	*n* = 82	*n* = 258	*n* = 76	*n* = 53	*n* = 47	*n* = 82
**Age (years)** **& Se serum (µg/L)**	***R* = 0.15** ***p* = 0.012**	***R*= −0.38** ***p* < 0.001**	***R* = 0.34** ***p* = 0.016**	*R* = 0.26 *p* = 0.06	*R* = 0.08 *p =* 0.46	*R= −0.02* *p =* 0.75	***R*= −0.25** ***p* = 0.03**	*R* = 0.18 *p =* 0.2	*R=* −0.15 *p =* 0.32	*R=* −0.17 *p =* 0.12
**Age (years)** **& Se urine (µg/L)**	***R* = 0.13** ***p* = 0.03**	*R=* −0.18 *p =* 0.07	***R* = 0.51** ***p* < 0.001**	***R* = 0.31** ***p* = 0.02**	*R* = 0.006 *p =* 0.96	*R* = 0.02 *p =* 0.73	***R*= −0.24** ***p* = 0.03**	*R* = −0.04 *p =* 0.78	*R* = 0.13 *p =* 0.39	***R* = 0.29** ***p* = 0.008**
**Age (years)** **& Se toenails (µg/g)**	***R* = 0.17** ***p* = 0.004**	*R* = 0.02 *p =* 0.84	*R=* −0.15 *p =* 0.3	***R* = 0.37** ***p* = 0.006**	*R=* −0.09 *p =* 0.44	*R* = 0.04 *p =* 0.5	*R* = 0.09 *p =* 0.42	*R* = −0.19 *p =* 0.18	*R* = 0.01 *p =* 0.97	***R*= −0.22** ***p* = 0.042**
**BMI (kg/m^2^)** **& Se serum (µg/L)**	***R* = −0.47** ***p* < 0.001**	***R* = −0.32** ***p* = 0.001**	***R* = 0.31** ***p* = 0.03**	***R* = −0.6** ***p* < 0.001**	*R* = 0.04 *p =* 0.68	***R* = −0.46** ***p* < 0.001**	***R* = −0.49** ***p* < 0.001**	*R* = 0.16 *p =* 0.26	***R* = −0.55** ***p* < 0.001**	***R* = −0.29** ***p* = 0.008**
**BMI (kg/m^2^)** **& Se urine (µg/L)**	***R* = −0.52** ***p* < 0.001**	***R* = −0.26** ***p* = 0.007**	*R* = 0.25 *p =* 0.08	***R* = −0.39** ***p* = 0.004**	*R* = −0.06 *p =* 0.61	***R* = −0.18** ***p* = 0.003**	***R* = −0.24** ***p* = 0.04**	*R* = 0.09 *p =* 0.5	*R* = −0.12 *p =* 0.43	*R=* −0.07 *p =* 0.51
**BMI (kg/m^2^)** **& Se toenails (µg/g)**	***R* = −0.51** ***p* < 0.001**	*R* = −0.04 *p =* 0.7	*R* = −0.19 *p =* 0.19	***R* = −0.29** ***p* = 0.03**	*R* = −0.05 *p =* 0.67	***R* = −0.52** ***p* < 0.001**	***R* = −0.35** ***p* = 0.002**	*R* = −0.11 *p =* 0.42	***R* = −0.54** ***p* < 0.001**	***R* = −0.25** ***p* = 0.02**
**TSH (µIU/mL) & Se serum (µg/L)**	***R* = −0.42** ***p* < 0.001**	*R* = 0.08 *p =* 0.43	*R* = −0.17 *p =* 0.24	***R* = −0.59** ***p* < 0.001**	*R* = −0.02 *p =* 0.82	***R*= −0.22** ***p* < 0.001**	***R*= −0.28** ***p* = 0.01**	***R* = 0.37** ***p* = 0.006**	***R*= −0.31** ***p* = 0.03**	*R* = 0.19 *p =* 0.08
**TSH (µIU/mL) &Se urine (µg/L)**	***R* = −0.38** ***p* < 0.001**	*R* = 0.0001 *p =* 0.999	*R* = −0.27 *p =* 0.06	***R* = −0.42** ***p* = 0.002**	*R* = −0.07 *p =* 0.51	***R* = −0.15** ***p* = 0.01**	*R* = −0.04 *p =* 0.74	*R* = 0.23 *p =* 0.09	*R* = −0.14 *p =* 0.33	*R* = 0.2 *p =* 0.07
**TSH (µIU/mL) & Se toenails (µg/g)**	***R* = −0.46** ***p* < 0.001**	*R* = −0.17 *p =* 0.094	*R* = −0.2 *p =* 0.17	***R* = −0.46** ***p* < 0.001**	*R* = −0.08 *p =* 0.48	***R* = −0.32** ***p* < 0.001**	***R* = −0.33** ***p* = 0.003**	*R* = −0.02 *p =* 0.9	***R* = −0.33** ***p* = 0.02**	*R* = 0.14 *p =* 0.22
**fT4 (pmol/L) & Se serum (µg/L)**	*R* = 0.04 *p =* 0.49	*R* = −0.02 *p =* 0.81	*R=* −0.1 *p =* 0.49	***R* = 0.39** ***p* = 0.004**	*R* = −0.04 *p =* 0.73	***R* = 0.13** ***p* = 0.04**	***R* = 0.28** ***p* = 0.01**	***R* = −0.29** ***p* = 0.034**	*R* = 0.14 *p =* 0.36	*R* = 0.06 *p =* 0.57
**fT4 (pmol/L) & Se urine (µg/L)**	*R* = 0.03 *p* = 0.63	*R* = −0.14 *p =* 0.16	*R* = −0.09 *p =* 0.55	*R* = 0.07 *p =* 0.63	*R* = 0.04 *p =* 0.75	*R* = 0.08 *p =* 0.19	*R* = 0.21 *p =* 0.07	*R* = −0.07 *p =* 0.61	*R* = −0.16 *p =* 0.28	*R* = 0.02 *p =* 0.88
**fT4 (pmol/L) & Se toenails (µg/g)**	*R* = 0.07 *p =* 0.23	*R* = −0.01 *p =* 0.9	*R* = 0.13 *p =* 0.38	***R* = 0.42** ***p* = 0.002**	*R* = 0.02 *p =* 0.84	***R* = 0.16** ***p* = 0.009**	***R* = 0.23** ***p* = 0.04**	*R* = 0.03 *p =* 0.85	*R* = 0.14 *p =* 0.35	*R* = −0.06 *p =* 0.57
**fT3 (pmol/L) & Se serum (µg/L)**	***R* = 0.23** ***p* < 0.001**	*R* = 0.06 *p =* 0.56	*R* = −0.1 *p =* 0.5	*R* = 0.16 *p* = 0.26	***R* = −0.23** ***p* = 0.04**	***R* = 0.15** ***p* = 0.02**	***R* = 0.26** ***p* = 0.02**	*R* = −0.17 *p =* 0.23	*R* = 0.22 *p =* 0.14	*R* = 0.15 *p =* 0.17
**fT3 (pmol/L) & Se urine (µg/L)**	***R* = 0.26** ***p* < 0.001**	*R* = −0.13 *p =* 0.17	*R* = 0.23 *p =* 0.11	*R* = 0.16 *p =* 0.24	*R* = 0.05 *p =* 0.66	***R* = 0.15** ***p* = 0.01**	*R* = 0.22 *p* = 0.06	*R* = 0.1 *p =* 0.47	*R* = 0.12 *p =* 0.42	*R* = 0.07 *p* = 0.54
**fT3 (pmol/L) & Se toenails (µg/g)**	***R* = 0.32** ***p* < 0.001**	*R* = 0.008 *p =* 0.94	*R* = −0.09 *p =* 0.52	***R* = 0.27** ***p* = 0.049**	***R* = 0.28** ***p* = 0.01**	***R* = 0.21** ***p* < 0.001**	***R* = 0.31** ***p* = 0.007**	*R* = −0.11 *p* = 0.43	*R* = 0.17 *p =* 0.25	*R* = 0.19 *p =* 0.09
**Se serum (µg/L)** **&Se urine (µg/L)**	***R* = 0.5** ***p* < 0.001**	***R* = 0.34** ***p* < 0.001**	***R* = 0.58** ***p* < 0.001**	***R* = 0.35** ***p* = 0.009**	*R* = 0.03 *p =* 0.76	***R* = 0.43** ***p* < 0.001**	***R* = 0.50** ***p* < 0.001**	***R* = 0.31** ***p* = 0.02**	***R* = 0.3** ***p* = 0.039**	***R* = 0.28** ***p* = 0.01**
**Se serum (µg/L)** **& Se toenails (µg/g)**	***R* = 0.56** ***p* < 0.001**	*R* = 0.12 *p* = 0.24	*R* = −0.13 *p =* 0.36	***R* = 0.27** ***p* = 0.049**	*R* = −0.011 *p =* 0.92	***R* = 0.49** ***p* < 0.001**	***R* = 0.26** ***p* = 0.02**	*R* = −0.19 *p =* 0.17	***R* = 0.59** ***p* < 0.001**	***R* = 0.59** ***p* < 0.001**
**Se urine (µg/L)** **& Se toenails (µg/g)**	***R* = 0.28** ***p* < 0.001**	*R* = −0.17 *p =* 0.09	*R* = −0.07 *p =* 0.64	*R* = 0.05 *p =* 0.71	*R* = 0.08 *p =* 0.46	***R* = 0.25** ***p* < 0.001**	*R* = 0.21 *p* = 0.07	*R* = 0.13 *p =* 0.36	*R* = 0.24 *p =* 0.10	*R* = 0.19 *p =* 0.08

**Table 3:** Inter-correlations between serum, urine and toenail selenium concentrations pre- and post-pubertally. Statistically significant correlations are highlighted. Spearman’s rank-order correlation test results.

	Total *n* = 258	ASD+/Ob+ *n* = 83	ASD+/Ob− *n* = 46	ASD−/Ob+ *n* = 51	ASD−/Ob− *n* = 78
Se serum before puberty (µg/L) & Se serum after puberty (µg/L)	***R*** **= 0.49** ***p*** **< 0.001**	***R*** **= 0.42** ***p*** **< 0.001**	***R*** **= 0.5** ***p*** **< 0.001**	*R* = 0.25 *p =* 0.08	*R* = 0.16 *p =* 0.16
Se urine before puberty (µg/L) & Se urine after puberty (µg/L)	***R*** **= 0.47** ***p*** **< 0.001**	***R*** **= 0.43** ***p*** **< 0.001**	***R*** **= 0.55** ***p*** **< 0.001**	***R*** **= 0.31** ***p*** **= 0.02**	***R*** **= 0.41** ***p*** **< 0.001**
Selenium toenails before puberty (µg/g) & Se toenails after puberty (µg/g)	***R*** **= 0.71** ***p*** **< 0.001**	*R* = 0.15 *p = 0.18*	***R*** **= 0.99** ***p*** **< 0.001**	***R*** **= 0.3** ***p*** **= 0.03**	***R*** **= 0.3** ***p*** **= 0.007**

**Table 4:** Multivariate linear regression analyses of serum, urine and toenail selenium concentrations (model adjusted for age, TSH, fT3, fT4) and logistic regression models of the associations between the presence of ASD, the presence of obesity/overweight, or gender, and serum, urine and toenail selenium concentrations.

Multivariate Linear Regression					
*n* = 258	BETA	SE ^a^ of BETA	B	SE ^a^ of B	*p*-Value
**Selenium Serum (µg/L)**					
ASD ^b^ (ASD 1, no ASD 0)	−0.15	0.05	−6.04	1.89	0.002
Gender (male 1; female 0)	−0.47	0.05	−20.33	2.04	<0.001
BMI ^c^ (kg/m^2^)	−0.39	0.05	−2.44	0.29	<0.001
**Selenium Urine (µg/L)**					
ASD ^b^ (ASD 1, no ASD 0)	−0.02	0.06	−0.37	1.36	0.79
Gender (male 1; female 0)	−0.34	0.06	−8.25	1.46	<0.001
BMI ^c^ (kg/m^2^)	−0.13	0.06	−0.44	0.21	0.03
**Selenium Toenails (µg/g)**					
ASD ^b^ (ASD 1, no ASD 0)	−0.39	0.05	−0.24	0.03	<0.001
Gender (male 1; female 0)	−0.26	0.05	−0.18	0.03	<0.001
BMI ^c^ (kg/m^2^)	−0.39	0.05	−0.04	0.004	<0.001
**Logistic Regression**					
***n* = 258**	**^d^ OR**	**^e^ 95% CI**			***p*-Value**
**^b^ ASD** (modeled probability that: ^b^ ASD 1, neurotypical 0 = 1)					
Selenium serum (µg/L)	0.97	0.95 to 0.99			0.003
Selenium urine (µg/L)	1.002	0.97 to 1.04			*0.* 87
Selenium toenails (µg/g)	0.0009	0.0001 to 0.006			<0.001
**Obesity/Overweight** (modeled probability that: obesity/overweight 1, normal weight 0 = 1)					
Selenium serum (µg/L)	0.95	0.93 to 0.97			<0.001
Selenium urine (µg/L)	1.002	0.98 to 1.03			0.86
Selenium toenails (µg/g)	0.01	0.003 to 0.046			<0.001
**Gender** (modeled probability that: male 1, female 0 = 1)					
Selenium serum (µg/L)	0.93	0.91 to 0.95			<0.001
Selenium urine (µg/L)	0.95	0.92 to 0.98			0.002
Selenium toenails (µg/g)	0.045	0.015 to 0.13			<0.001

^a^ SE—standard error; ^b^ ASD—autism spectrum disorder; ^c^ BMI—body mass index;^d^ OR—odds ratio; ^e^ CI—confidence interval.

## Abbreviations

ASD	Autism Spectrum Disorder
SE	Selenium
PDD-NOS	Pervasive Developmental Disorder Not Otherwise Specified
AS EDCs	Asperger Syndrome Endocrine disrupting chemicals
DSMV	Diagnostic and Statistical Manual of Mental Disorders, Fifth Edition
ADI-R	Autism Diagnostic Interview Revised
CARS	Autism Rating Scale
BMI	Body Mass Index
fT3	Free Triiodothyronine
fT4	Free Thyroxine
TSH	Thyroid Stimulating Hormone
ICP-MS	Inductively Coupled Plasma Mass Spectrometry
IQR	Interquartile Range
SD	Standard Deviation
SE	Standard Error
OR	Odds Ratio
CI	Confidence Interval
